# Cytotoxic effects and the mechanism of three types of magnetic nanoparticles on human hepatoma BEL-7402 cells

**DOI:** 10.1186/1556-276X-6-480

**Published:** 2011-07-29

**Authors:** Wei Kai, Xu Xiaojun, Pu Ximing, Hou Zhenqing, Zhang Qiqing

**Affiliations:** 1 Department of Chemistry, College of Chemistry and Chemical Engineering, Xiamen University, Xiamen 361005, PR China; 2 Research Center of Biomedical Engineering, Department of Materials Science and Engineering, College of Materials, Xiamen University, Technology Research Center of Biomedical Engineering of Xiamen City, The Key Laboratory of Biomedical Engineering of Fujian Province, Xiamen 361005, PR China; 3 Zhejiang Fishery Technical Extention Center, Hangzhou 310012, PR China; 4 Institute of Biomedical Engineering, Chinese Academy of Medical Science and Peking Union Medical College, The Key Laboratory of Biomedical Material of Tianjin, Tianjin 300192, PR China

**Keywords:** magnetic nanoparticles, BEL-7402, apoptosis, mitochondrial-dependent pathway, cell cycle

## Abstract

The evaluation of the toxicity of magnetic nanoparticles (MNPs) has attracted much attention in recent years. The current study aimed to investigate the cytotoxic effects of Fe_3_O_4_, oleic acid-coated Fe_3_O_4 _(OA-Fe_3_O_4_), and carbon-coated Fe (C-Fe) nanoparticles on human hepatoma BEL-7402 cells and the mechanisms. WST-1 assay demonstrated that the cytotoxicity of three types of MNPs was in a dose-dependent manner. G1 (Fe_3_O_4 _and OA-Fe_3_O_4_) phase and G2 (C-Fe) phase cell arrests and apoptosis induced by MNPs were detected by flow cytometry analysis. The increase in apoptosis was accompanied with the Bax over-expression, mitochondrial membrane potential decrease, and the release of cytochrome C from mitochondria into cytosol. Moreover, apoptosis was further confirmed by morphological and biochemical hallmarks, such as swollen mitochondria with lysing cristae and caspase-3 activation. Our results revealed that certain concentrations of the three types of MNPs affect BEL-7402 cells viability via cell arrest and inducing apoptosis, and the MNPs-induced apoptosis is mediated through the mitochondrial-dependent pathway. The influence potency of MNPs observed in all experiments would be: C-Fe > Fe_3_O_4 _> OA-Fe_3_O_4_.

## Introduction

Over the past few decades, as nanotechnology and materials science has progressed incredibly swiftly, nanomaterials have been widely applied in many fields including medicine, pharmaceuticals, manufacturing technologies, electronics, and telecommunications [1-3]. In particular, the surge of interest in nanomaterials has significantly expanded the breadth of research on magnetic nanoparticles (MNPs) during the recent decade. Due to their multifunctional properties, MNPs are explored for various biomedical applications such as contrast agents for MRI [4,5], targeted drug and gene delivery [6,7], cell sorting [8], hyperthermia [9], or combinations of multiple applications, both diagnostic and therapeutic [10]. Some MNPs, such as bowel contrast agents (Lumiren^® ^and Gastromark^®^) and liver/spleen imaging (Endorem^® ^and Feridex IV^®^) [11,12], are already in the market. Moreover, the potential applications of MNPs (*e.g*., bare Fe_3_O_4 _and C-Fe) have expanded into other fields including environmental restoration [13,14] and agriculture [15-18]. Some researches indicate that MNPs would accumulate in aquatic organisms [19], crops [18] for further entry into the food chain. Humans are therefore increasingly exposed to various kinds of MNPs, directly or indirectly.

Along with the expanding applications of MNPs, the potential toxic effects of MNPs have been of wide concern [20-23]. Multiple results show that MNPs significantly reduce cell viability of human macrophage, epithelial cell lines [24], human mesothelioma [25], and inhibit the normal formation of PC12 neuronal cell morphology [26]. At higher concentrations, DMSA-coated MNPs decrease mitochondrial activity of human fibroblasts [27]. Meanwhile, the cytotoxicity of MNPs is found in a dose-dependent manner [26].

Nevertheless, the cytotoxicity data of MNPs is difficult to compare since the toxic effects of MNPs are influenced by many parameters such as size distribution, surface coating, magnetic properties, *etc*. [27]. Numerous studies can be found that, quite often, report on seemingly contradicting findings since different cell types will interact with the same particle in different ways [28]. Therefore, it is crucial to choose the cell line for the cytotoxicity assessment of specific MNPs. Several pharmacokinetic reports indicate that liver is the most important organ involving the bioaccumulation and clearance procedures of MNPs [29-31]. Furthermore, the cytotoxicity studies of MNPs are limited by the fact that cytology mechanism remained unexplored.

In the present study, human hepatoma BEL-7402 cell line was selected as the model specimen for cytotoxicity assessment, and the aims were to evaluate the cytotoxicity of Fe_3_O_4_, OA-Fe_3_O_4_, and C-Fe and to elucidate the mechanisms of their cytotoxicities. MNPs internalization was observed by transmission electron microscopy (TEM) and cell viability was determined by tetrazolium salt-based (WST-1) assay. For the study of the mechanism of cytotoxicity, cell cycle and apoptosis were analyzed by flow cytometry. To further elucidate the apoptosis pathway, the mitochondrial membrane potential (MMP), the Bax and cytochrome C protein expression, and caspase-3 activity were investigated.

## Results and discussion

### MNPs uptake by human hepatoma BEL-7402 cells

**Figure 1:**
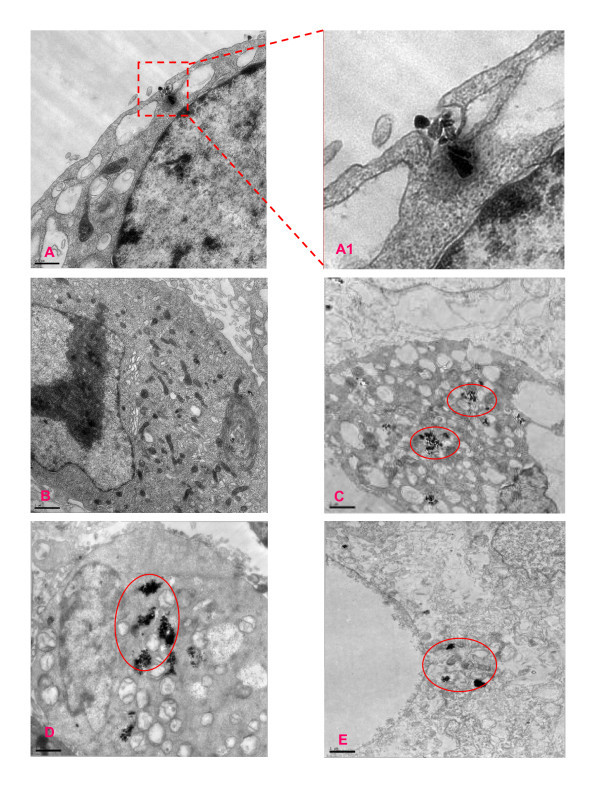
**TEM micrographs of BEL-7402 exposed for 24 h to the different MNPs**. (**A**) TEM micrographs of cell endocytosis; (**A1**) the enlargement of the rectangular areas on the corresponding images on the left side; (**B**) untreated cells; (**C**) cells exposed 0.5 mg/mL Fe_3_O_4 _MNPs; (**D**) cells exposed 0.5 mg/mL OA- Fe_3_O_4 _MNPs; (**E**) cells exposed 0.5 mg/mL C-Fe MNPs. Red circles show MNPs in the BEL-7402 cells.

When cells were exposed to MNPs, most nanoparticles were first adhered to the surface, internalized to the cells by endocytosis, and accumulated in digestive vacuoles [32]. Our TEM images results showed that all three kinds of MNPs were incorporated into BEL-7402 cells after 24-h incubation at 0.5 mg/mL of concentration. The MNPs were distributed on the cell membrane and inside of cell. Some MNPs were observed enclosed by the invaginated cell membrane (Figure 1A), suggesting that endocytosis may involve the MNPs internalization process [21]. Lysosomes containing MNPs and swollen mitochondria with lysing cristae were present in MNPs treated cells (Figure 1C-D), coinciding with some results obtained in other MNPs [21,33]. Some cells showed chromatin condensation, typical of apoptotic cell death, and plenty of cytoplasmic vacuoles (Figure 1C-E). Treating with OA-Fe_3_O_4 _induced less cell damage than that of Fe_3_O_4_, while the C-Fe causes serious cell damage. Untreated cells had none of these features (Figure 1B).

### The dose-dependent cytotoxicity of nanoparticles

**Figure 2:**
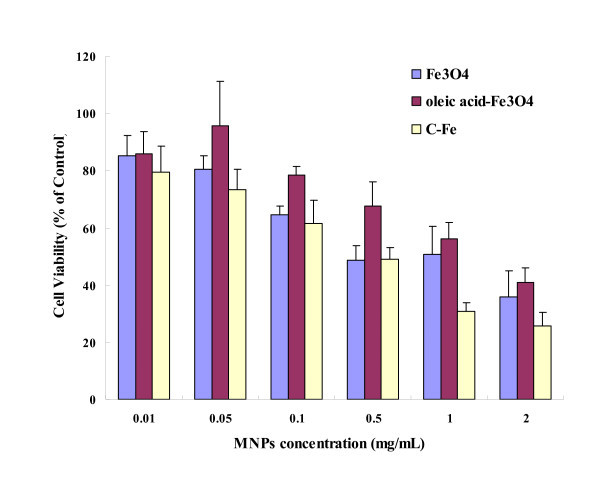
**The viability of BEL-7402 Cells incubated with MNPs**. Cells viability was determined by WST-1 assay after BEL-7402 cells were treated with MNPs (0.01, 0.05, 0.1, 0.5, 1, and 2 mg/mL) for 24 h. The percentage of viable cells was calculated as a ratio of absorbance at 490 nm of treated to control cells.

3-(4, 5-Dimethylthiazol-2-yl)-2, 5-diphenyltetrazolium bromide and lactate dehydrogenase assays are frequently adopted in assessing nanoparticle toxicity. These assays are used in drug studies, but can lead to aberrant results when using nanoparticles as they can sometimes interfere with the assay components or the readout [34]. Due to its convenience and great sensitivity, recently, the WST-1 assay has become a very popular cytotoxicity assay in the nanotoxicity study [22]. After 24 h exposure at varying doses of Fe_3_O_4_, OA-Fe_3_O_4_, and C-Fe MNPs, BEL-7402 cell viabilities detected by the WST-1 assay resulted in explicit dose-dependent reduction (Figure 2). The viabilities of BEL-7402 cells exposed to all three types of MNPs were above 60% at the concentration of 0.1 mg/mL and below. When the MNPs concentrations increased more than 1 mg/mL, the cell viabilities dropped to below 60%. The viabilities of cells exposed to Fe_3_O_4 _were lower than that to OA-Fe_3_O_4_, but higher than to C-Fe at all concentrations, which were correlated with the TEM observations. The cytotoxicity is thus very likely caused by particle overload to cells [35]. It is well known that the surface of BEL-7402 cells is negatively charge. The MNPs absorbed by the cells reduced with the decrease in positively charged surfaces of MNPs due to the electrostatic effects, which could affect the amount of MNPs entering the cells and further affect cytotoxicity. In our results, the surface charge of Fe_3_O_4_, OA-Fe_3_O_4_, and C-Fe were 14.4, 4.5, and 23.7 mV, respectively, which were consistent with WST-1 data trend.

### MNPs influence on the cell cycle

The effects of various concentrations (0.05, 0.1, and 1 mg/mL) of the three kinds of MNPs on cell cycle progression and population distribution in BEL-7402 cells were determined by flow cytometry. MNPs-induced effects were detected by comparing the cell cycle profiles between MNP-treated and -untreated cells. Results demonstrated that all three types of MNPs were able to affect cell cycle distribution of BEL-7402 cells (Table 1). After treated with Fe_3_O_4 _and OA-Fe_3_O_4 _MNPs at three different concentrations (0.05, 0.1, and 1 mg/mL), the ratio of G0/G1 phase cells increased 3.42%, 18.70%, 28.78% and 4.37%, 3.46%, 15.71% compared with control, respectively. As for C-Fe, 2.85%, 3.21%, and 9.34% G2 phase cell increases were observed. A similar report also showed that single-walled carbon nanotubes also caused a G2 phase arrest in PC12 cells [36]. Therefore, the mechanism of C-Fe MNPs on the cell cycle might be different with that of Fe_3_O_4_, and OA-Fe_3_O_4 _MNPs.

**Table 1:** MNPs affected cell cycle distribution of BEL-7402 cells

	Cell cycle (%)			
		**G0-G1**	**S**	**G2**

Control		60.13	32.65	7.22
Fe_3_O_4_	0.05 mg/mL	63.55	26.72	9.73
	0.1 mg/mL	78.83*	14.09*	7.08
	1 mg/mL	88.91	3.52**	7.57
OA-Fe_3_O_4_	0.05 mg/mL	64.50	30.37	5.13
	0.1 mg/mL	63.59	29.75	6.67
	1 mg/mL	75.84*	21	3.15
C-Fe	0.05 mg/mL	59.32	30.61	10.07
	0.1 mg/mL	65.96	23.61	10.43
	1 mg/mL	56.56	26.88	16.56*

After BEL-7402 cells were treated with MNPs (0.05 and 1 mg/mL) for 24 h, cell cycle assay was carried out by PI staining using flow cytometry. *P < 0.05 *vs*. control; **P < 0.01 *vs*. control.

Cells with reversibly damaged DNA will accumulate in G1, S, or G2/M phase [36], while cells that carry irreversibly damaged DNA will undergo apoptosis [37,38]. Hence, it is necessary to further analyze the cell apoptosis to fully interpret the toxic effects of MNPs on BEL-7402 cells.

### MNPs-induced apoptosis of BEL-7402 cells

**Figure 3:**
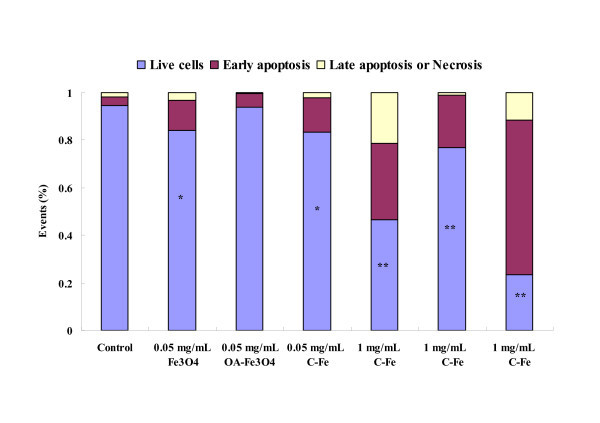
**MNPs induced apoptosis of BEL-7402 cells by Annexin V/PI assay**. Annexin V-FITC/PI assay shows cell apoptosis by flow cytometry. Exposure of BEL-7402 cells to MNPs (0.05 and 1 mg/mL) for 24 h increased cell apoptosis. *P < 0.05 *vs*. control; **P < 0.01 *vs*. control.

To assess the extent and mode of cell death induced by MNPs, Annexin-V/propidium iodide (PI) staining was performed. Externalization of phosphatidylserine (PS) seems to be a general feature of early stage apoptosis. Annexin V which has a strong Ca^2+^-dependent affinity for PS [39] was used to measure the apoptotic rate of BEL-7402 cells in response to the treatment of MNPs. The BEL-7402 cells were labeled with annexin V-fluorescein isothiocyanate (FITC)/PI. The Annexin V^-^/PI^- ^population was regarded as normal cells, while positive staining just for Annexin V was used as a measure of early apoptosis and Annexin V^-^/PI^+ ^was related to late apoptosis or necrosis [40]. Statistical data were extracted from the dot plots using WinMDI software [37]. As shown in Figure 3, compared with the untreated cells, a significant increase in the ratio of apoptosis cell was observed in Fe_3_O_4 _and C-Fe MNPs (0.05 mg/mL) treated cells (*P *< 0.05, the probability values of *P *< 0.05 were considered as statistics significance). At high concentration (1 mg/mL), all MNPs cause serious cell apoptosis (*P *< 0.01). Besides, a dose-dependent apoptosis rate was observed in all three types of MNP-treated cells. Moreover, the apoptosis rate of cells exposed to three types of MNPs would be: C-Fe > Fe_3_O_4 _> OA-Fe_3_O_4_, in the same concentration.

This apoptosis result is consistent with cytotoxicity trends shown in WST-1 assay. The mechanisms of cytotoxic effects of MNPs on BEL-7402 cells may be implemented through cell cycle arrest and inducing apoptosis.

### Assay of mitochondria-dependent apoptosis in BEL-7402 cells after MNPs

Apoptosis is a tightly controlled process in which cell death is executed through the activation of specific signaling pathways [41,42]. Although it is well established that many organelles contribute to apoptosis, extensive research shows that nanoparticles induced cell apoptosis via mitochondria-dependent pathway [43,44]. As an indicative of mitochondria involvement in the apoptosis, the apparently swollen mitochondria with lysing cristae were observed by TEM (Figure 1). Therefore, we speculate that BEL-7402 cell apoptosis was induced by MNPs through mitochondria-dependent pathway.

**Figure 4:**
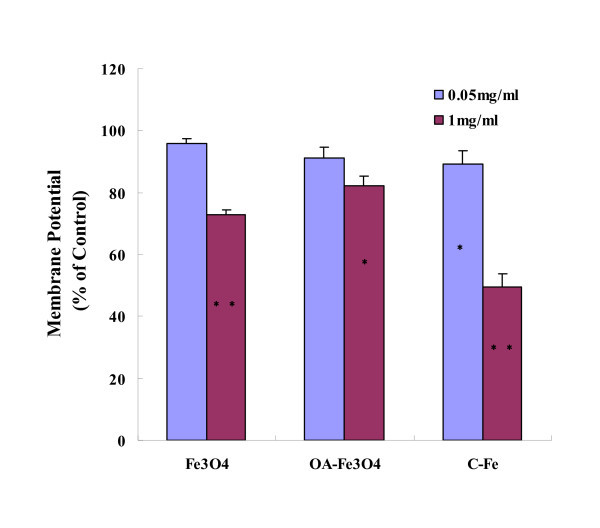
**MNPs-induced loss of MMP**. The MMP was measured by flow cytometry using JC-1 dye. Exposure of BEL-7402 cells to three types of MNPs (0.05 and 1 mg/mL) for 24 h decreased the MMP. *P < 0.05 *vs*. control; **P < 0.01 *vs*. control.

The mitochondrion is an important organelle involved in apoptosis. The loss of MMP is putatively the initial event leading to apoptosis [45]. To further elucidate the molecular mechanism of MNPs-induced apoptosis in BEL-7402, we examined loss of MMP using flow cytometry. As illustrated in Figure 4, after 24-h exposure to MNPs (0.05 mg/mL) for 24 h, a significant decrease in MMP was only observed in C-Fe-treated group (*P *< 0.05), while at high concentration (1 mg/mL), significant decrease of MMP occurred in all three MNPs-treated groups (*P *< 0.05).

**Figure 5:**
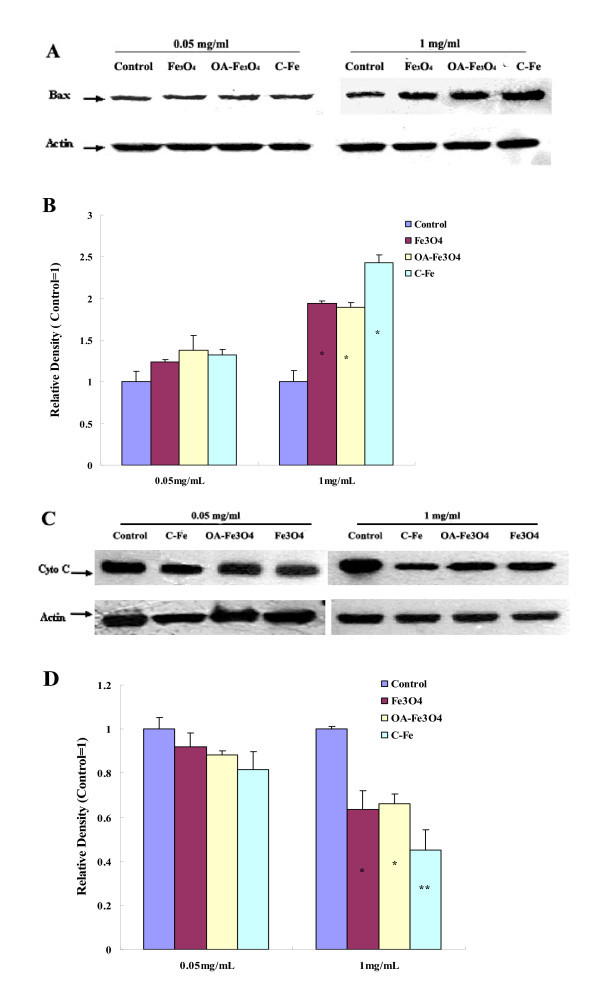
**MNPs induced Bax over-expression and Cytochrome C release**. Up-regulation of Bax expression in BEL-7402 cells treated with MNPs (**A**) and plotted as a relative level (**B**); down-regulation of cytochrome C expression in BEL-7402 cells treated with MNPs (**C**) and plotted as a relative level (**D**) *P < 0.05 *vs*. control; **P < 0.01 *vs*. control.

**Figure 6:**
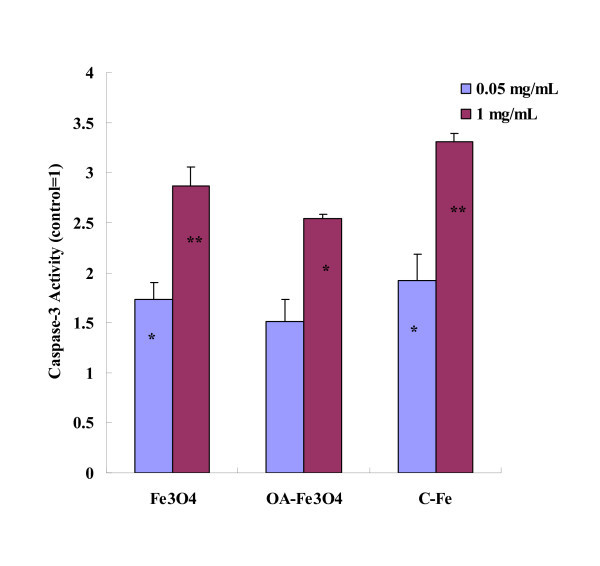
**Caspase-3 activity of BEL-7402 cells after incubation with MNPs for 24 h**. After BEL-7402 cells were treated with MNPs (0.05 and 1 mg/mL) for 24 h, caspase-3 activity was determined using caspase-3/CPP32 Colorimetric Assay Kit. *P < 0.05 *vs*. control; **P < 0.01 *vs*. control.

Mitochondrial membrane permeability is regulated through a family of proto-oncogenes. Bax is an important pro-apoptotic protein of the Bcl-2 family members [46]. High level of Bax can translocate to the outer mitochondrial membrane (OMM) and insert into the OMM. Then, Bax forms oligomers that are thought to be important in the formation of the mitochondrial permeability transition pore (PTP) [47,48]. The opening of the mitochondrial PTP can lead to a release of cytochrome C, which is a key event in apoptosis via the mitochondria-mediated pathway [49]. We examined expression of Bax and cytochrome C by Western blot. As shown in Figure 5A-B, after 24 h exposure at low concentration (0.05 mg/mL) of MNPs, the expression of Bax protein slightly increased, while without significant differences (*P *> 0.05). At high concentration (1 mg/mL), Bax protein expression in Fe_3_O_4_, OA-Fe_3_O_4_, and C-Fe MNPs-treated groups were about 1.94, 1.89, and 2.43 times compared with the control group, respectively. As for cytochrome C, the protein expression slightly decreased at the low concentration, while without significant differences (*P *> 0.05). At high concentration, the cytochrome C protein expression of all treated groups decreased dramatically, which is consistent with tendency of MMP. Based on the results mentioned above, we concluded that the three types of MNPs could induce Bax expression, further open PTP, and the PTP opening led to the release of cytochrome C from mitochondria. Once released from the mitochondria, cytochrome C combines with procaspase-9 to form the "apoptosome", which further activates caspase-3 [50-52]. Caspase-3 has been identified as a key mediator of apoptosis of mammalian cells [53]. Its activity is considered to be an appropriate measure of cytotoxic responsiveness [54]. We investigated the activaty of caspases-3 in BEL-7402 after exposure to MNPs for 24 h. As shown in Figure 6, we found that all three types of MNPs can activate caspase-3 in a dose-dependent manner. At low concentration (0.05 mg/mL), the activity of caspase-3 of the experimental groups increased, with significant differences found in Fe_3_O_4_- and C-Fe-treated groups (*P *< 0.05). The activity of caspase-3 was significantly increased in all experimental groups at high concentration (1 mg/mL) (*P *< 0.05).

To sum up, our results indeed suggested that all three types of MNPs can induce apoptosis in BEL-7402 cells through mitochondria-dependent pathway. Moreover, the influence potency of MNPs on the mitochondria-dependent apoptosis would be: C-Fe > Fe_3_O_4 _> OA-Fe_3_O_4_, and all in a dose-dependent manner.

## Conclusion

In this paper, cytotoxic effects and the mechanism of Fe_3_O_4_, OA- Fe_3_O_4_, and C-Fe MNPs on BEL-7402 cells were studied. A dose-dependent cytotoxicity pattern was found in all three types of MNPs via WST-1 assay. The results of flow cytometric analysis revealed that the cytotoxicity of MNPs is implemented through cell cycle arrest and inducing apoptosis. The results of mitochondrial membrane potential, Western blots for Bax and cytochrome C, and caspase-3 activation further elucidate that MNPs induce apoptosis through mitochondria-dependent pathway. Moreover, the influence potency of MNPs observed in all experiments would be: C-Fe > Fe_3_O_4 _> OA-Fe_3_O_4_.

Recent studies show that the cytotoxicities of many MNPs could be due to reactive oxygen species (ROS) induction [55,56]. And accompanied with the MNPs degradation, the altered cellular iron pool can then affect cellular functionality by altering the level of transferrin receptor expression and can affect cellular proliferation capacity by altering the expression of cyclins and cyclin-dependent kinases in cell cycle [57,58]. Therefore, the metabolism, ROS determination and transferrin receptor expression will be the next step for further reveal of the cytotoxicities of Fe_3_O_4_, OA- Fe_3_O_4_, and C-Fe.

## Materials and methods

### Reagents

RPMI-1640 and fetal bovine serum were purchased from Gibco, Invitrogen Corp., Carlsbad, CA, USA. PI and RNase I were obtained from Sigma, St. Louis, MO, USA. Alexa Fluor^® ^488 annexin V/Dead Cell Apoptosis Kit was obtained from Invitrogen, USA. The primary antibodies to Bax, cytochrome C, and β-actin were purchased from Santa Cruz Biotechnology (Santa Cruz, CA, USA). The goat anti-Mouse IgG-HRP, mouse anti-rabbit IgG-HRP, and Potent ECL kit were purchased from Multisciences, Hanzhou, China. Caspase-3/CPP32 Colorimetric Assay Kit and Mitochondria/Cytosol Fractionation Kit were purchased from BioVision, Mountain View, CA, USA. Total Protein Extraction Kit and BCA Protein Assay Kit were obtained from Applygen Technologies Inc., Beijing, China. The lipophilic cationic dye JC-1 (5, 5, 6, 6-tetrachloro-1, 1, 3, 3-tetraethylbenzimidazol-carbocyanine iodide) was obtained from ChemoMetec, Allerød, Denmark. WST-1 Cell Proliferation and Cytotoxicity Assay Kit was purchased from Beyotime Institute of Biotechnology, Haimen, China. All other reagents are analytical or cultured grade purity.

### Cell culture and preparation of MNPs

Human hepatoma BEL-7402 cell line was a gift kindly provided by Medical College of Xiamen University (Xiamen, China). The cells were cultured in RPMI-1640 medium supplemented with 10% heat-inactivated fetal bovine serum. Incubation was carried out at 37°C in a humidified 5% CO_2 _incubator. For all experiments, the cells were in the exponential growth phase. The MNPs used in this study were: (1) Fe_3_O_4 _MNPs, purchased from Aladdin (Shanghai, China); (2) OA-Fe_3_O_4 _MNPs, purchased from Jinke (Maanshan, China); (3) C-Fe MNPs, purchased from Junye (Shenzhen, China). The purity of three types MNPs are 99.9% and the size distribution of particles are 10-30 nm. Nanoparticle stock suspensions (10 mg/mL) were prepared by UV-sterilization and dispersing a known weight of nanoparticles in RPMI-1640 medium under ultrasonication. The stock suspensions were sonicated for 20 min to distribute the particles, and then dilutions were made in complete media to achieve desired testing concentrations. The test suspensions were sonicated for 20 min before use. Untreated controls were exposed to complete media only, and processed identical to the exposed cells.

### TEM analysis

Cells (2 × 10^6^) were seeded into 100-cm^2 ^petri dishes. Cells were allowed to attach for 24 h and were then treated with each MNPs test suspensions for 24 h in a concentration of 0.5 mg/mL. Then, the cells were collected and fixed with 2.5% glutaraldehyde buffered in 0.1 M PBS overnight at 4°C. The samples were washed with PBS, and post-fixed in 1% osmium tetroxide at 4°C for 1 h. After dehydration in series concentrations of ethanol and infiltration in acetone, cells were embedded in Epon 812, and ultra-thin sections cut with glass knives were stained with uranyl acetate and lead citrate, and viewed under JEM 2100 TEM (JEOL, Tokyo, Japan).

### WST-1 assay

To determine cell toxicity/viability, BEL-7402 cells (0.5 × 10^4^, 100 μL) were plated onto 96-multiwell plates (Costar, Corning, NY, USA) and incubated for 24 h. Then, cells were exposed to various concentrations (0.01-2 mg/mL) of each MNPs test suspensions for 24 h. Afterwards, the old media was discarded and replaced with 100 μL of new complete media. WST-1 solution (10 μL) was added to each well, followed by incubation for 2.5 h. Absorbance at 490 nm (reference at 630 nm) was measured by a spectrophotometric microplate reader (Bio-tek ELX800, BioTek Instruments, Winooski, VT1, USA). A negative control was provided using the culture medium without the nanoparticles. Each of the particle concentrations and the controls was seeded in eight wells. The percentage cell viability was calculated in term of absorbency in cells treated with MNPs relative to that in cells exposed to culture media alone.

### Cell cycle assay

A cell cycle assay was carried out by staining the DNA with PI and analyzing the fluorescence using flow cytometry. Following exposure of the BEL-7402 cells to each MNPs for 24 h, any damaged cells can detach from the plate and become suspended in the medium, necessitating medium storage. Briefly, the cells were harvested, washed with PBS, and fixed in ice-cold 70% of ethanol at -20°C before use. After resuspension, cells were washed, and incubated with 100 μL PI (400 μg/mL) and 100 μL RNase I (1 mg/mL) at 37°C for 15 min. Cells were analyzed with an EPICS XL flow cytometer (Beckman Coulter Inc., Fullerton, CA, USA) and the data were consequently evaluated by Mod-Fit (Verity Software, Topsham, ME, USA).

### Detection of apoptosis by annexin V assay

Apoptosis was evaluated using Alexa Fluor^® ^488 Annexin V/PI Apoptosis Kit. BEL-7402 cells were treated with two concentrations (0.05 and 1 mg/mL) of each MNPs for 24 h. After exposure, the cells (5-10 × 10^4^) were harvested, washed and resuspended with PBS. The Annexin V/PI staining of cells followed the manufacturer's instructions. Then the samples were analyzed with EPICS XL flow cytometer (Beckman Coulter, USA). The results were expressed as the number of apoptotic cells per thousand cells counted.

### MMP measurement

The mitochondrial membrane potential was measured by flow cytometry using JC-1 dye. JC-1 changes its fluorescence from green at 535 nm (monomer state) to orange at 590 nm (aggregate state) as it enters in mitochondria of intact cells. When the mitochondrial membrane potential is affected, JC-1 returns to its green monomeric state. All procedures were carried out according to the manufacturer's instructions. The cell treatments were the same with "Detection of apoptosis by annexin V assay" section. In brief, approximately 2 × 10^6 ^cells were harvested, washed, resuspended in PBS (1 mL) and stained with 12.5 μL JC-1(200 μg/mL) for 15 min at 37°C in the dark. Both fluorescences emitted by the cells were monitored by flow cytometry and the ratio orange/green fluorescence was calculated. The data was determined by analyzing 10,000 cells using an EPICS XL flow cytometer (Beckman Coulter, Fullerton, CA, USA), and Cell Quest software (Becton Dickinson, San Jose, CA, USA).

### Western blot analysis of Bax and cytochrome C

The cell treatments were the same with "Detection of apoptosis by annexin V assay" section. Approximately 1 × 10^7 ^cells per sample were harvested. The protein samples of Bax and cytochrome C were extracted using Total Protein Extraction Kit and Mitochondria/Cytosol Fractionation Kit, respectively. Protein contents were quantified using the BCA protein assay kit and stored at -70°C. Protein (20 μg per lane) was resolved by 12% sodium dodecyl sulfate-polyacrylamide gel electrophoresis (SDS-PAGE), and transferred to nitrocellulose membranes (PVDF, Millipore Corporation, Billerica, MA, USA). The transblotted membrane was washed, blocked, and incubated at 4°C overnight with anti-Bax antibody and anti-cytochrome C antibody, respectively. Immunodetection with the secondary HRP-conjugated antibody and chemiluminescence using Potent ECL Kit were performed according to the manufacturer's protocol. Equal protein loading was verified by probing with anti-β actin antibody. Densitometric analysis for the blots was performed with Bandscan image software.

### Caspase-3 activity assay

The activity of caspase-3 was determined using caspase-3/CPP32 Colorimetric Assay Kit. The cell treatments were the same with "Detection of apoptosis by annexin V assay" section. All procedures were carried out according to the manufacturer's instructions. Briefly, 2 × 10^7 ^BEL-7402 cells were lysed by the solution provided in the assay kit and the protein concentration was measured using BCA protein assay kit. For caspase-3 activity assay, equal amounts of total cell lysates were mixed with a caspase-specific substrate DEVD-pNA in a 96-well plate in triplicate. After incubation at 37°C for 2 h, the caspase-3-mediated cleavage of DEVD-pNA into free pNA was measured using spectrophotometric microplate reader (Bio-tek ELX800, USA) at 405 nm. The results were expressed as absorbance compared with control.

### Statistical analysis

All results were expressed as mean values ± S.D. Statistical analysis was performed according to the Student's *t *test. The probability values of *P *< 0.05 were considered as significant.

## Competing interests

The authors declare that they have no competing interests.

## Authors' contributions

WK conceived the study, carried out all the experiments and drafted the manuscript. XXJ collected and analysed data, drafted the manuscript and approved the final version. PXM participated in drafting the manuscript. HZQ reviewed the manuscript. ZQQ conceived the study, reviewed the manuscript and approved the final version.
